# Draft genome sequence of *Xylaria* sp., the causal agent of taproot decline of soybean in the southern United States

**DOI:** 10.1016/j.dib.2017.12.060

**Published:** 2018-01-03

**Authors:** Sandeep Sharma, Alex Z. Zaccaron, John B. Ridenour, Tom W. Allen, Kassie Conner, Vinson P. Doyle, Trey Price, Edward Sikora, Raghuwinder Singh, Terry Spurlock, Maria Tomaso-Peterson, Tessie Wilkerson, Burton H. Bluhm

**Affiliations:** a Department of Plant Pathology, University of Arkansas, Fayetteville, AR 72701, United States; b Mississippi State University, Delta Research and Extension Center, Stoneville, MS 38776, United States; c Alabama Cooperative Extension System, Auburn University, AL 36849, United States; d Department of Plant Pathology and Crop Physiology, LSU AgCenter, Baton Rouge, LA 70803, United States; e Macon Ridge Research Station, LSU AgCenter, Winnsboro, LA 71295, United States; f Auburn University, Auburn, AL 36849, United States; g Department of Plant Pathology, University of Arkansas System Division of Agriculture, Southeast Research and Extension Center, Monticello, AR 71656, United States; h Mississippi State University, MS 39762, United States

## Abstract

The draft genome of *Xylaria* sp. isolate MSU_SB201401, causal agent of taproot decline of soybean in the southern U.S., is presented here. The genome assembly was 56.7 Mb in size with an L50 of 246. A total of 10,880 putative protein-encoding genes were predicted, including 647 genes encoding carbohydrate-active enzymes and 1053 genes encoding secreted proteins. This is the first draft genome of a plant-pathogenic *Xylaria* sp. associated with soybean. The draft genome of *Xylaria* sp. isolate MSU_SB201401 will provide an important resource for future experiments to determine the molecular basis of pathogenesis.

**Specifications Table**

**Table:** 

Subject area	Biology
More specific subject area	Plant Pathology, Mycology, Genomics
Type of data	Genomic sequence, gene prediction and annotation of *Xylaria* sp. isolate MSU_SB201401
How data was acquired	Whole genome was sequenced with an Ion Torrent PGM
Data format	Draft genome assembly and gene annotation
Experimental factors	The fungus was cultured on yeast extract peptone dextrose medium at room temperature, and DNA was extracted with a modified CTAB protocol
Experimental features	The genome was assembled with SPAdes version 3.6 and annotated with MAKER version 2.31.6
Data source location	Isolate MSU_SB201401 was collected from diseased soybean roots in Louisiana, USA; genome sequencing was performed at the University of Arkansas, USA
Data accessibility	The whole genome shotgun project of *Xylaria* sp. isolate MSU_SB201401 has been deposited at DDBJ/ENA/GenBank under the accession number NPFG00000000; the version described in this paper is version NPFG01000000

**Value of the data**

• The first draft genome of *Xylaria* sp. isolate MSU_SB201401, a causal agent of taproot decline on soybean, is presented.
• Mechanisms of pathogenesis in the genus *Xylaria* are poorly understood.
• The draft genome will accelerate functional genomics research and help to understand the molecular basis of pathogenicity.

## 1 Data

The genus *Xylaria* (Xylariales, Xylariaceae) is the largest genus in the family Xylariaceae. The genus is mostly comprised of saprophytes associated with dead organic matter and endophytes commonly associated with crops like soybean and barley [1]. Morphological features of the genus include large stromatal tissue containing multiple perithecia, cylindrical asci, and dark ascospores with a germ slit [2]. Interestingly, few species of *Xylaria* have been reported as pathogens [3]. Plant-pathogenic species of *Xylaria* include *Xylaria mali*, the causal agent of root rot of apple in the southern and midwestern United States [4], and *Xylaria arbuscula* associated with quick decline of *Macadamia* trees [5]. Recently, a taxonomically unresolved member of *Xylaria* was reported as the causal organism for taproot decline of soybean in the southern U.S., especially along the Mississippi River Valley [6]. Initial phylogenetic analyses of the isolate with four nuclear loci placed the isolate within the *Xylaria arbuscula* species aggregate [6]. Soybean plants affected by taproot decline often exhibit interveinal chlorosis in the leaves of the lower canopy during vegetative growth stages, which increases in severity as plants mature through reproductive stages of growth and can be observed in the upper canopy during advanced reproductive growth stages (>R6). Taproots and lateral roots display a blackened appearance, with stroma of the fungus frequently visible upon examination. The disease causes premature plant death in severe cases [6].

Taproot decline is the first report of a soybean disease caused by a species of *Xylaria*. However, this was not the first reported observation of this particular fungal genus associated with soybean. Isolation of an unknown species of *Xylaria* from soybean seed that had originated in Ethiopia was previously reported [7], but that isolate was not confirmed to be a pathogen of soybean. To accelerate taxonomic resolution, determination of molecular mechanisms underlying pathogenesis, functional genomics research, and the development of molecular detection assays for the pathogen, the genome of *Xylaria* sp. isolate MSU_SB201401 associated with taproot decline was sequenced and assembled. DNA sequencing with the Ion Torrent PGM platform generated 7,099,268 reads with a mean read length of 281 bp and a total of 2 Gb of data. The resulting genome assembly of *Xylaria* sp. MSU_SB201401 contained 56.9 Mb, which was notably larger than the 42.8 Mb genome of *Xylaria hypoxylon* (http://genome.jgi.doe.gov/Xylhyp1/Xylhyp1.home.html) and the 40.9 Mb genome of *Xylaria* sp. JS573 (GenBank accession JWIU00000000.1). The genome assembly of *Xylaria* sp. MSU_SB201401 consisted of 6629 contigs (4743 longer than 1 kb), with an N50 of 56 kb, and an L50 of 246. The longest contig spanned 423,920 bp. The GC content of the genome was predicted to be 43.3%. Approximately 14.3% of the genome assembly was comprised of repetitive elements. Transposable elements comprised 7.8% of the genome assembly, with the retrotransposon family TY1_Copia being the most prevalent (6.2% of the genome assembly). A total of 10,881 genes were predicted (average length=1694 bp), encoding proteins with an average length of 496 amino acids. BUSCO analyses of the predicted genes showed 94.7% completeness with 0.3% duplication, 4.0% fragmentation, and 1.3% of genes missing. Of the 10,881 predicted proteins, 10,678 (98%) had at least one homologous sequence in the NCBI nr database (e-value<1e-5). Blast2GO attributed a GO term for 9076 (83%) of the proteins, and 6674 (61%) were functionally annotated. A total of 647 genes encoding carbohydrate-active enzymes (CAZymes) were identified, which included 279 glycoside hydrolases (GHs), 91 glycosyltransferases (GTs), 17 polysaccharide lyases (PLs), 109 carbohydrate esterases (CEs), and 137 auxiliary activities (AAs). Additionally, the genome of *Xylaria* sp. MSU_SB201401 was predicted to contain 1053 genes encoding secreted proteins, which included 314 CAZymes, and 96 proteases.

Although *Xylaria* spp. commonly exist as wood decomposers or endophytes, there are limited reports of pathogenic species within the genus. Thus, mechanisms underlying pathogenesis among species of *Xylaria* are largely unknown. In the context of fundamental research, the genome assembly of *Xylaria* sp. isolate MSU_SB201401 presented herein will advance the discovery of molecular mechanisms underlying taproot decline of soybean and accelerate taxonomic resolution of the causal organism. In an applied context, the draft genome assembly of *Xylaria* sp. isolate MSU_SB201401 will facilitate the development of rapid detection assays for the pathogen in plant and environmental samples. Together, these efforts will ultimately help provide important management tools for taproot decline of soybean.

## 2 Experimental design, materials and methods

### 2.1 Genomic DNA extraction and sequencing

Three mycelial plugs (3–5 mm^2^ each) from *Xylaria* sp. isolate MSU_SB201401 grown on V8 juice agar medium were transferred into yeast extract peptone dextrose medium [8] amended with 100 μg ml^−^^1^ carbenicillin (Research Products International, Mt. Prospect, IL, USA) and incubated at room temperature with constant shaking at 100 rpm for 5 days. Fungal tissue was collected by centrifugation at 4000 rpm for 5 min and ground with liquid nitrogen. Genomic DNA was extracted as previously described [9]. The quality and quantity of the genomic DNA were assessed by agarose gel electrophoresis and spectrophotometric analysis, respectively. A 400 bp library was prepared from genomic DNA with the NEBNext Fast DNA Fragmentation and Library Preparation Kit (New England Biolabs, Ipswich, NE, USA) following the manufacturer's instructions. Library quality was evaluated with Agilent Tapestation D1000 tape (Agilent Technologies, Santa Clara, CA, USA). Whole genome sequencing was performed at the University of Arkansas, Fayetteville, AR, USA, with an Ion Torrent Personal Genome Machine (PGM) (Thermo Fisher Scientific, Waltham, MA, USA) and a 318 V2 chip kit.

### 2.2 Genome assembly and annotation

The genome was assembled with SPAdes version 3.6 [10] with default parameters for Ion Torrent reads. Protein-encoding genes were predicted with the MAKER pipeline version 2.31.6 [11], with support from *ab initio* predictors SNAP version 2006-07-28 [12] and Augustus version 3.0.2 [13]. Proteins from *Xylaria hypoxylon* OSC 100004 and *Hypoxylon* sp. CO27-5 (accessed from http://genome.jgi.doe.gov/programs/fungi/index.jsf) and *Daldinia* sp. EC12 (GenBank accession MDGZ00000000.1) were also used to support gene prediction, by providing protein homology evidence. Gene completeness of the draft assembly was assessed with Benchmarking Universal Single-Copy Orthologs (BUSCO) software version 2 [14] based on the Ascomycota dataset. The predicted protein sequences were used as queries in a local BLAST search (BLAST version 2.2.31) against the NCBI nr database (update 02/2016). For functional annotation, BLAST search results were analyzed with Blast2GO version 4.1 [15]. CAZymes were identified with dbCAN version 5.0 [16]. Proteins predicted to contain a signal peptide by both SignalP version 4.1 [17] and TargetP version 1.1 [18] were categorized as secreted proteins. Proteases were identified by homology (BLAST) searches against the MEROPS database version 11.0 [19] with a maximum e-value of 1e-5.
